# Stem Cell Mobilization Performed with Different Doses of Cytarabine in Plasma Cell Myeloma Patients Relapsing after Previous Autologous Hematopoietic Cell Transplantation—A Multicenter Report by the Polish Myeloma Study Group

**DOI:** 10.3390/cancers16142588

**Published:** 2024-07-19

**Authors:** Joanna Drozd-Sokołowska, Anna Waszczuk-Gajda, Magdalena Topczewska, Martyna Maciejewska, Magdalena Dutka, Jan Maciej Zaucha, Anna Szmigielska-Kapłon, Mateusz Nowicki, Magdalena Olszewska-Szopa, Agnieszka Szeremet, Anna Czyż, Magdalena Kozioł, Marek Hus, Joanna Mańko, Iwona Hus, Joanna Romejko-Jarosińska, Anna Kopińska, Grzegorz Helbig, Krzysztof Mądry, Piotr Boguradzki, Małgorzata Król, Emilian Snarski, Patrick J. Hayden, Krzysztof Jamroziak, Jadwiga Dwilewicz-Trojaczek, Grzegorz Władysław Basak

**Affiliations:** 1 Department of Hematology, Transplantation and Internal Medicine, Medical University of Warsaw, 02-097 Warsaw, Poland; anna.waszczuk-gajda@wum.edu.pl (A.W.-G.); martyna.maciejewska@uckwum.pl (M.M.); kmadry@wum.edu.pl (K.M.); piotr.boguradzki@wum.edu.pl (P.B.); malgorzata.krol@uckwum.pl (M.K.); emiliansnarski@gmail.com (E.S.); k.m.jamroziak@gmail.com (K.J.); jadwiga.dwilewicz-trojaczek@wum.edu.pl (J.D.-T.); grzegorz.basak@wum.edu.pl (G.W.B.); 2 Faculty of Computer Science, Bialystok University of Technology, 15-351 Bialystok, Poland; m.topczewska@pb.edu.pl; 3 Department of Hematology and Transplantology, Medical University of Gdansk, 80-214 Gdansk, Poland; magdalena.dutka@gumed.edu.pl (M.D.); jan.zaucha@gumed.edu.pl (J.M.Z.); 4 Department of Hematology, Medical University of Lodz, 93-513 Lodz, Poland; aszmigielska@poczta.onet.pl (A.S.-K.); mat.nowicki@gmail.com (M.N.); 5 Department of Hematology, Wroclaw Medical University, 50-367 Wroclaw, Poland; molszopa@gmail.com (M.O.-S.); agnieszka.szeremet@wp.pl (A.S.); aczyz@onet.eu (A.C.); 6 Department of Hematooncology and Bone Marrow Transplantation, Medical University of Lublin, 20-081 Lublin, Poland; magkoziol@op.pl (M.K.); marekhus@umlub.pl (M.H.); joanna_m@go2.pl (J.M.); iwonach.hus@gmail.com (I.H.); 7 Department of Hematology, National Medical Institute of the Ministry of Interior and Administration, 02-507 Warsaw, Poland; 8 Department of Lymphoid Malignancies, Maria Sklodowska-Curie National Research Institute of Oncology, 02-781 Warsaw, Poland; joanna.romejko-jarosinska@pib-nio.pl; 9 Department of Hematology and Bone Marrow Transplantation, Medical University of Silesia, 40-032 Katowice, Poland; cauda.equina@wp.pl (A.K.); ghelbig@o2.pl (G.H.); 10 Faculty of Medicine and Health Science, University of Zielona Góra, 65-046 Zielona Góra, Poland; 11 Department of Haematology, Trinity College Dublin, St. James’s Hospital, D08 NHY1 Dublin, Ireland; phayden@stjames.ie

**Keywords:** stem cell mobilization, autologous hematopoietic cell transplantation, plasma cell myeloma, relapse, salvage treatment

## Abstract

**Simple Summary:**

Autologous hematopoietic cell transplantation (auto-HCT) can be used to salvage at least a proportion of plasma cell myeloma patients who relapse after a previous auto-HCT. It may, however, occur that there is either no or an insufficient stem cell dose in storage to proceed to transplantation. Remobilization to procure new cells is then required. There are very limited data in the literature concerning the efficacy of stem cell remobilization after previous auto-HCT. In our previous report, we showed that remobilization with cytarabine was associated with a lower risk of remobilization failure in comparison to etoposide or cyclophosphamide. In the current study, we analyze the efficacy and safety of different doses of cytarabine (800, 1600, and 2400 mg/m^2^), showing that all doses are efficacious but that the dose of 2400 mg/m^2^ is associated with the most toxicity. Therefore, lower doses of cytarabine seem to be preferable, with plerixafor rescue when needed.

**Abstract:**

Salvage autologous hematopoietic cell transplantation (auto-HCT) may be used to treat relapse of plasma cell myeloma occurring after previous auto-HCT. When an insufficient number of hematopoietic stem cells have been stored from the initial harvest, remobilization is necessary. Here, we aimed to analyze the efficacy and safety of different doses of cytarabine (total 800 vs. 1600 vs. 2400 mg/m^2^) for remobilization. Sixty-five patients, 55% male, with a median age at remobilization 63 years, were included. Remobilization was performed with cytarabine_800 in 7, cytarabine_1600 in 36, and cytarabine_2400 in 22 patients. Plerixafor rescue was used in 25% of patients receiving cytarabine_1600 and 27% of those receiving cytarabine_2400. Patients administered cytarabine_800 were not rescued with plerixafor. Remobilization was successful in 80% of patients (57% cytarabine_800; 86% cytarabine_1600; 77% cytarabine_2400; *p* = 0.199). The yield of collected CD34+ cells did not differ between the different cytarabine doses (*p* = 0.495). Patients receiving cytarabine_2400 were at the highest risk of developing severe cytopenias, requiring blood product support, or having blood-stream infections. One patient died of septic shock after cytarabine_2400. In summary, remobilization with cytarabine is feasible in most patients. All doses of cytarabine allow for successful remobilization. Cytarabine_2400 is associated with higher toxicity; therefore, lower doses (800 or 1600 mg/m^2^) seem to be preferable.

## 1. Introduction

Autologous hematopoietic cell transplantation (auto-HCT) is considered the standard of care for eligible patients diagnosed with plasma cell myeloma (PCM) [1]. It is typically performed in the upfront setting for newly diagnosed PCM. Auto-HCT can also be performed following relapse, including patients who relapse after a prior auto-HCT(s), as has been shown in both prospective and retrospective studies [2,3,4,5,6]. It is recommended that the relapse-free interval after the first auto-HCT(s) has been sufficiently long, at least 18 months if not on any treatment, or at least 36 months if the patient has been on maintenance lenalidomide [7,8,9], to justify proceeding to salvage auto-HCT [1].

In patients considered likely to benefit from a salvage transplant, only some will have stem cells in storage. If not, remobilization will be required.

There are limited data in the literature concerning the efficacy of stem cell remobilization after previous myeloablative treatment [10,11,12,13,14,15,16], and there is no consensus on the optimal remobilization protocol. Data on the efficacy and safety of salvage auto-HCT performed with remobilized stem cells [17,18] are scarce. In our previous report performed under the auspices of the Polish Myeloma Study Group, we showed that remobilization with cytarabine was associated with a lower risk of remobilization failure in comparison to etoposide or cyclophosphamide [15]. Here, we report an analysis of remobilization performed with a range of cytarabine doses and provide guidance on optimal remobilization dosing strategies.

## 2. Materials and Methods

### 2.1. Data Source

The study was performed on behalf of the Polish Myeloma Study Group, a voluntary organization comprising hematology and oncology centers in Poland that provides care for PCM patients [19]. All member centers were invited to participate in this study and provide additional study-specific data about eligible patients.

### 2.2. Study Population and Outcome

The study was approved by the Ethical Board of the Medical University of Warsaw (Approval ID: AKBE/141/2024) and was performed in accordance with the Declaration of Helsinki. Patients gave informed consent for treatment and follow-up analysis.

This retrospective study included PCM patients who underwent either single or tandem auto-HCT(s) and subsequently relapsed and who, during salvage treatment, underwent remobilization of stem cells with cytarabine. The remobilization procedures were performed between 2010 and 2021. Seven centers participated in the study. All consecutive patients undergoing remobilization were included in this analysis. Analysis was performed on both the total patient population and on the three different cytarabine dosing cohorts.

The primary endpoint was the efficacy of different doses of cytarabine for remobilization. Mobilization failure was defined as a collection < 2 × 10^6^ CD34+ cells/kg body weight, the lowest CD34+ cell dose considered acceptable for an autologous transplant [20]. The secondary endpoint was the comparative safety of the three cytarabine doses.

PCM staging was based on the International Staging System (ISS) [21], while the response to treatment was assessed according to the International Myeloma Working Group [22]. Toxicity was graded according to Common Terminology Criteria for Adverse Events (CTCAE) version 5.0 [23].

### 2.3. Remobilization

The dose of cytarabine was chosen at the discretion of the treating physician or center policy. Cytarabine was always used with granulocyte colony-stimulating factor (G-CSF) either alone or in combination with plerixafor. G-CSF was administered either at a daily dose of 10 µg/kg body weight divided into two doses or 5 µg/kg body weight once daily until the ninth day of the procedure and 10 µg/kg body weight thereafter. Plerixafor was administered at a dose of 240 µg/kg body weight in patients with an estimated glomerular filtration rate of more than 50 mL/min. Cytarabine was administered as a 2 h infusion at a dose of 400 mg/m^2^ twice daily on days 1 and 2 (total dose 1600 mg/m^2^) or 1 through 3 (total dose 2400 g/m^2^). Patients administered a total dose of 800 mg/m^2^ received cytarabine as a 2 h infusion at a dose of 400 mg/m^2^ once daily on days 1 and 2.

The day of initiation of G-CSF was centre-dependent. The general policy was to start on either Day +5 or Day +7. The duration of G-CSF administration varied based on local policy. In line with reimbursement criteria, plerixafor was added when, despite the use of an adequate mobilization regimen, the maximum CD34+ cell count in the peripheral blood was <10/μL in the first 20 days. It could also be used in the setting of prior mobilization failures defined as a collection of <2 × 10^6^ CD34+ cells/kg body weight when a single auto-HCT was planned or <4 × 10^6^ CD34+ cells/kg body weight when a tandem auto-HCT was intended. The threshold to start leucapheresis was a CD34+-cell count of at least 10/μL (and preferentially > 20/μL), and collections were performed using either the Spectra-Optia Apheresis System (CaridianBCT Inc., Lakewood, CO, USA) or ComTec (Fresenius Kabi, Bad Homburg vor der Höhe, Germany).

Supportive treatment, including febrile neutropenia prophylaxis, anti-bacterial, antifungal, and anti-viral prophylaxis, antimicrobial therapy, and blood product support, were administered based on local policy.

### 2.4. Statistical Analysis

Data on all study-eligible patients were collected using a standardized, anonymized case report form and included patient and disease characteristics at baseline, treatment, remobilization-specific data, and toxicities. Data were reviewed by the coordinating investigator for consistency and, if necessary, queries resolved with local clinicians.

Patient-, disease-, and remobilization-related variables were expressed as median and range for continuous variables and frequencies and percentages (of all patients with data available) for categorical variables.

Differences between groups were compared using the Kruskal–Wallis equality-of-populations rank test if the assumption of distributions’ normality was violated.

Logistic regression was performed to assess the relationship between the likelihood of remobilization failure and patient-, disease- and remobilization-specific variables. The results are presented as odds ratios (OR) along with 95% confidence intervals (CI) and are significant for *p* < 0.05. All calculations were performed using Stata/IC ver 11.0 (StataCorp LLC, College Station, TX, USA).

## 3. Results

### 3.1. Patients

Sixty-five patients were included in the analysis. There were 36 (55%) men, and the median age at remobilization was 63 years (range, 37–71). The median calendar year of remobilization was 2017 (range 2010–2021). The monoclonal protein was IgG in 62% of cases. Most patients had advanced-stage PCM at diagnosis (ISS stage III—39/59, 66%). Fifty-two patients (80%) had previously had a single auto-HCT, while thirteen (20%) had had tandem auto-HCTs. The first reinduction regimen at relapse after a previous auto-HCT was bortezomib-based in most patients (44, 68%). Thirty-six out of sixty-four patients (56%) achieved a very good partial remission (VGPR) or better after salvage treatment and before remobilization. The median interval between the most recent auto-HCT and remobilization was 42 months (range 8–239). The median number of lines of therapy the patients had received prior to remobilization was two (range 1–6). For further details, please see Table 1 and Supplementary Table S1.

### 3.2. Dose of Cytarabine and Efficacy of Remobilization

The dose of cytarabine used for the first remobilization was 800 mg/m^2^ in 7 patients (11%), 1600 mg/m^2^ in 36 (55%), and 2400 mg/m^2^ in 22 (34%). Patients remobilized with cytarabine_2400 were treated during earlier calendar years (median 2015 vs. 2018 for both cytarabine_1600 and cytarabine_800). In addition, patients remobilized with cytarabine_2400 were more likely to have had disease progression at the time of remobilization than patients remobilized with either cytarabine_1600 or cytarabine_800 (32% vs. 0% vs. 0%, respectively; *p* = 0.001).

The first remobilization attempt resulted in a successful collection of ≥2 × 10^6^ CD34+ cells/kg body weight in 52 patients (80%).

Among the seven patients remobilized with cytarabine_800, five (71%) patients started leukapheresis, but only four (57%) collected ≥ 2 × 10^6^ CD34+ cells/kg body weight. Plerixafor rescue, according to the center policy, was not used in patients with mobilization failure. Two patients who successfully collected stem cells were harvested in one apheresis procedure. Thirty-three (92%) patients remobilized with cytarabine_1600 started leukapheresis, and thirty-one (86%) had a total yield of ≥2 × 10^6^ CD34+ cells/kg body weight. Plerixafor rescue was used in nine (25%) patients and was effective in seven (78%). Cytarabine_2400 allowed for successful collection in 17 (77%) patients; plerixafor was used in 6 (27%) individuals and was effective in 5 (83%). Precise data on the efficacy of different doses of cytarabine are presented in Table 2.

The total yield of collected CD34+ cells/kg did not differ significantly between the three cytarabine doses, and the median number of cells was 5.4 × 10^6^/kg vs. 4.9 vs. 7.5 for cytarabine_800, cytarabine_1600 and cytarabine_2400, respectively.

### 3.3. Toxicity of Subsequent Cytarabine Dosesat Remobilization

The use of cytarabine_2400 was associated with profound cytopenias (thrombocytopenia Grade 3 or 4–91%; neutropenia Grade 3 or 4–86%). For patients remobilized with cytarabine_1600, the respective frequencies were 55% and 43%, while for cytarabine_800–80% and 25%, respectively. Platelet transfusions were mostly administered to patients remobilized with cytarabine_2400 (18 patients, 82%; *p* = 0.003), with the median number of days requiring transfusions being two (range, 1–9). Patients remobilized with cytarabine _1600 required platelet transfusions in 37% of cases, the median number of days being one (range, 1–3), while patients remobilized with cytarabine_800 required platelet transfusions in 33% of patients. Grade 4 neutropenia developed most frequently in patients remobilized with cytarabine_2400 (77% vs. 29% vs. 0%; *p* < 0.001). Patients receiving cytarabine_2400 also spent a longer period with neutropenia, both <0.5 and 0.5–1.0 × 10^9^/L. Though anemia occurred in all patients, patients remobilized with cytarabine_2400 required the most red blood cell transfusions (55% vs. 22% vs. 14%; *p* = 0.021).

The other common side effects were infections, reported in 10% of patients remobilized with cytarabine_1600 and 41% of patients remobilized with cytarabine_2400. The most frequent were blood-stream infections (27% among patients receiving cytarabine_2400). Pneumonia occurred in one patient each in the cytarabine_2400 and the cytarabine_1600 groups. Patients receiving cytarabine_2400 required antibiotics (usually piperacillin–tazobactam and/or aminoglycosides) significantly more frequently than patients in other groups. For detailed information on the site of the infection, see Table 3. One patient remobilized with cytarabine_2400 died of septic shock caused by *Escherichia coli*.

### 3.4. Factors Predictive for Mobilization Failure

Univariate logistic regression analysis was performed to identify factors associated with remobilization failure. Only a platelet count ≤ 100 × 10^9^/L was associated with an increased risk of remobilization failure, with an OR = 6.13 (95% CI, 1.05 to 35.82). The other factor of borderline significance was older age (>65 years), which was again associated with a trend to a higher rate of mobilization failure (OR = 3.16, 95% CI 0.91 to 11.11). The dose of cytarabine did not affect the efficacy of remobilization in the analyzed cohort. Detailed data on the impact of other potential predictive factors is presented in Table 4.

## 4. Discussion

The treatment landscape of relapsed/refractory (r/r) PCM is constantly evolving, with new agents and combinations of agents being approved each year. Modern therapies like CAR T cells (chimeric antigen receptor T cells) and bispecifics (e.g., teclistamab, elranatamab, talquetamab) are gradually being made available in earlier lines of therapy, as reviewed in [24]. Despite all this progress, PCM remains an incurable disease, and more effective salvage therapies are needed. Another obstacle in the treatment of r/r PCM is the lack of availability of expensive novel therapies, which remain largely unavailable in resource-poor regions. Therefore, the role of auto-HCT remains, and it may be used to treat a proportion of r/r PCM patients [6,7,8,9,25,26].

As mentioned earlier, the availability of stem cells remains a challenge in patients considered suitable for salvage auto-HCT after prior transplantation. In the absence of a stored product, remobilization to procure new cells is required. This was infrequently undertaken in the past because patients who had undergone myeloablative therapy were considered likely to be poor mobilizers [27,28]. We have previously shown, however, that remobilization performed with the use of chemotherapy in such patients is successful in 67% of patients in general and that the efficacy is dependent on the choice of chemotherapy, cytarabine being associated with the most efficacy (84% vs. 53% for cyclophosphamide vs. 55% for etoposide) [15]. This is not surprising given the efficacy of cytarabine in the first- or second-line setting [29,30,31,32]. Our current report confirms that cytarabine is a very effective drug for remobilization, with success rates reaching 80%. The doses utilized in the current study were either 1600 mg/m^2^, as proposed by Kruzel et al. [31]; 2400 mg/m^2^, as described by Montillo et al. [33]; or 800 mg/m^2^, as proposed by Snarski et al. [34]. Importantly, cytarabine was effective regardless of the dose, and the success rate of remobilization was 57% for cytarabine_800, 86% for cytarabine_1600, and 77% for cytarabine_2400; *p* = 0.199. Admittedly, plerixafor rescue was used in 25% of patients receiving cytarabine_1600 and 27% receiving cytarabine_2400. Based on our local policy, it was not administered to patients receiving cytarabine_800 because of the high expected success rate in a general PCM population, as reported in [34]. As we have shown, plerixafor rescue was effective in 77% of patients in the cytarabine_1600 and 78% in the cytarabine_2400 cohorts, which is consistent with reports on the efficacy of plerixafor ranging between 55% and 82% of proven or predicted poor mobilizers [13,28,35,36]. In our study, the efficacy of plerixafor was among the highest reported to date.

The efficacy of remobilization of 80% is impressive. As a comparison, the success rate of the first remobilization attempts in the study of Parish et al., where most patients were remobilized with cyclophosphamide + G-CSF, or G-CSF alone, was only 37.3%, and 49.1% after all remobilization attempts [10]. In the study of Baertsch et al., the efficacy of high-dose cyclophosphamide-based remobilization was comparable to the efficacy of cytarabine in our study, though 56% of these patients who were successfully remobilized required plerixafor rescue (in comparison to 25–27% of the patients in our study) and two out of the thirty patients in their report died of mobilization-associated septic shock [14].

Despite the fact that the dose of cytarabine did not affect the efficacy of remobilization, it did affect the toxicity. The most frequent adverse events observed were cytopenias and infections. Grade 3/4 anemia and thrombocytopenia occurred at similar rates in patients treated with different doses of cytarabine. Nonetheless, the severity of thrombocytopenia and anemia was greater in those patients receiving cytarabine_2400, and significantly more patients required blood product support (55% packed red blood cells, 82% platelets transfusions). In addition, patients receiving cytarabine_2400 experienced more severe neutropenia and spent significantly more days being neutropenic than patients receiving lower doses of cytarabine. The rate of blood-stream infections (BSI) was also highest among patients receiving cytarabine_2400, resulting in much more frequent use of antimicrobial agents (41%). The only remobilization-associated death was in a patient receiving cytarabine_2400 who died of septic shock complicating *Escherichia coli* BSI. We cannot exclude the possibility that higher toxicity of cytarabine_2400 was at least partially associated with a higher rate of progressive disease at remobilization in this group of patients.

In our study, we did not analyze patients who were remobilized with single agent G-CSF or G-CSF in combination with plerixafor. It is recognized that chemotherapy-based mobilization is associated with more toxicity. However, the use of chemotherapy also has the potential to increase the efficacy of mobilization and to increase the yield of CD34+ cells [11,37,38].

Logistic regression analysis allowed for the identification of thrombocytopenia ≤ 100 × 10^9^/L as a factor associated with poorer remobilization efficacy (OR = 6.125, 95% CI 1.047 to 35.824). This phenomenon was previously identified by Papanikolaou et al. [11], who also identified the use of single agent growth factors without chemotherapy, pre-collection hemoglobin < 110 g/L, female sex, and albumin < 35 g/L as other poor prognostic factors. In our study, although we did not perform any genetic/ molecular analysis, it is possible that thrombocytopenia may be a surrogate for clonal hematopoiesis (CH) with accompanying cytopenias, i.e., clonal cytopenia of unknown significance (CCUS). Further support for this hypothesis comes from the study of Papanikolaou, in which anemia was associated with poorer remobilization efficacy. In our study, older patients (>65 years) had a trend to a higher rate of remobilization failure, which again could support this CH hypothesis, i.e., age-related clonal hematopoiesis (ARCH) [39].

The number of collected CD34+ cells/ kg did not differ between the different doses of cytarabine. It should, however, be kept in mind that the decision to stop collection could have been made based on the yield of stem cells. It can be assumed that, most probably, once a sufficient dose of CD34+ cells to facilitate an auto-HCT had been collected, the remobilization was stopped so as to minimize the costs and risks associated with the procedure itself. Therefore, a simple comparison of CD34+ cell yield is probably not the ideal method to establish the optimum cytarabine dose.

There are some important limitations to our study. First, it is a retrospective study, and the number of patients is limited. Secondly, the changing first-line treatment regimens, the availability of maintenance, and the availability of novel drugs to treat relapse were not considered in this analysis. Third, we did not perform genetic/ mutational studies to check for clonal hematopoiesis. Nevertheless, we provide robust data to demonstrate that remobilization with cytarabine is very effective.

## 5. Conclusions

To conclude, remobilization with cytarabine is effective, regardless of the dose. Higher doses of cytarabine are, however, associated with greater toxicity and both hematological and infectious complications. Therefore, it is reasonable to conclude that either a dose of 1600 m/m^2^ or 800 mg/m^2^ total is preferable, with plerixafor rescue when needed.

## Figures and Tables

**Table 1:** Patients’ characteristics (auto-HCT—autologous hematopoietic cell transplantation; CTD—cyclophosphamide, thalidomide, dexamethasone; CR—complete remission; PCM—plasma cell myeloma; VGPR—very good partial remission; PR—partial remission; SD—stable disease; PD—progressive disease).

	Total	Cyatarabine_800	Cytarabine_1600	Cytarabine_2400	*p*
Number of patients	65	7	36	22	-
Calendar year of remobilization; median (range)	2017 (2010–2021)	2018 (2018–2019)	2018 (2012–2021)	2015 (2010–2021)	0.0001
Age at remobilization; years, median (range)	63 (37–71)	68 (46–70)	60 (42–71)	63 (37–68)	0.179
Sex					0.505
Male	36 (55%)	4 (57%)	22 (61%)	10 (45%)	
Female	29 (45%)	3 (43%)	14 (39%)	12 (55%)	
Total number of lines of therapy; median (range)	2 (1–6)	2 (2–5)	2 (1–5)	2 (1–6)	0.962
Radiotherapy used at any time prior to remobilization	9 (14%)	1 (14%)	4 (11%)	4 (18%)	0.751
First reinduction treatment for relapse					0.390
CTD	5 (8%)	0 (0%)	1 (3%)	4 (18%)	
Bortezomib-based	44 (68%)	5 (71%)	27 (75%)	12 (55%)	
Other	14 (22%)	2 (29%)	7 (19%)	5 (23%)	
No treatment	2 (3%)	0 (0%)	1 (3%)	1 (5%)	
Drugs used anytime for PCM treatment prior to remobilization					
Alkylators	51 (78%)	7 (100%)	27 (75%)	17 (77%)	0.334
Bortezomib	58 (89%)	7 (100%)	34 (94%)	17 (77%)	0.077
Carfilzomib	4 (6%)	2 (29%)	2 (6%)	0 (0%)	0.023
Thalidomide	56 (86%)	7 (100%)	31 (86%)	18 (82%)	0.479
Lenalidomide	17 (26%)	3 (43%)	12 (36%)	2 (9%)	0.071
Pomalidomide	0 (0%)	0 (0%)	0 (0%)	0 (0%)	-
Bendamustine	2 (3%)	0 (0%)	2 (6%)	0 (0%)	0.436
Daratumumab	2 (3%)	0 (0%)	2 (6%)	0 (0%)	0.436
Time interval between the most recent auto-HCT and remobilization; months, median (range)	42 (8–239)	66 (14–76)	36 (9–239)	49 (8–169)	0.154
Platelet count at the start of remobilization; ×10^9^/L, median (range)	184 (61–395) (missing: 2)	161 (61–298) (missing: 1)	183 (72–395) (missing: 1)	210 (78–286)	0.308
Platelet count ≤ 150 × 10^9^/L at the start of remobilization	15 (24%) (missing: 2)	2 (33%) (missing: 1)	11 (31%) (missing: 1)	2 (9%)	0.132
Platelet count ≤ 100 × 10^9^/L at the start of remobilization	6 (10%) (missing: 2)	1 (17%) (missing: 1)	4 (11%) (missing: 1)	1 (5%)	0.567

**Table 2:** Efficacy of remobilization in general and comparison between subsequent doses of cytarabine used for remobilization (G-CSF—granulocyte colony-stimulating factor).

	Total	Cyatarabine_800	Cytarabine_1600	Cytarabine_2400	*p*
Number of patients	65	7	36	22	-
Number of patients who started leukapheresis	59 (91%)	5 (71%)	33 (92%)	21 (95%)	0.154
Number of patients who collected ≥ 2 × 10^6^ CD34+ cells/kg body weight	52 (80%)	4 (57%)	31 (86%)	17 (77%)	0.199
Number of patients who collected ≥ 2 × 10^6^ CD34+ cells/kg body weight during one procedure of leukapheresis	46 (71%)	4 (57%)	26 (72%)	16 (73%)	0.703
Number of remobilizations with a total yield of (2 × 10^6^ CD34+ cells/kg body weight according to the number of previous auto-HCTs					
1	42/52 (81%)	3/4 (75%)	30/35 (86%)	9/13 (69%)	0.417
2	10/13 (77%)	1/3 (33%)	1/1 (100%)	8/9 (89%)	0.120
Total number of collected CD34+ cells; ×10^6^/kg body weight *; median, range	5.76 (0.65–33.86)	5.36 (1.52–9.07)	4.89 (1.35–33.86)	7.5 (0.65–18.49)	0.495
Maximal number of CD34+ cells/μL; median (range)	61.9 (0–1860) (missing: 24)	15 (1–144) (missing: 2)	61 (0–1860) (missing: 10)	80.6 (9–822.3) (missing: 8)	0.364
Start of leukapheresis; day *; median (range)	16 (5–24)	14 (14–20)	16 (5–22)	16 (5–24)	0.494
Number of leukapheresis procedures; median (range)	1.5 (1–4)	2 (1–2)	1 (1–4)	2 (1–4)	0.956
Start day of G-CSF; day, median (range)	6 (3–14)	5 (5–7)	6.5 (3–14)	6.5 (3–8)	0.383
Plerixafor rescue	15 (23%)	0 (0%)	9 (25%)	6 (27%)	0.302
Successful plerixafor rescue	12 (80%)	-	7 (78%)	5 (83%)	1.0

* Solely for patients who collected any cells/initiated leukapheresis.

**Table 3:** Toxicity of subsequent doses of cytarabine used for remobilization (BSI—blood-stream infection; CVC—central venous catheter; RBCs—red blood cells; PLTs—platelets; URTI—upper respiratory tract infection; UTI—urinary tract infection).

	Total	Cyatarabine_800	Cytarabine_1600	Cytarabine_2400	*p*
Number of patients	65	7	36	22	-
Death associated with remobilization	1 (2%)	0 (0%)	0 (0%)	1 (4.5%)	0.371
Anemia, number of patients, %					
Any grade	53 (100%)	4 (100%)	27 (100%)	22 (100%)	-
Grade 3/4	14 (26%) (missing: 12)	1 (25%) (missing: 3)	8 (30%) (missing: 9)	5 (23%)	0.860
Number of patients requiring RBC transfusion	21 (32%)	1 (14%)	8 (22%)	12 (55%)	0.021
Number of transfused RBC units, median, range	2 (1–10)	2	2 (1–4)	2 (2–10)	0.247
Thrombocytopenia, number of patients, %					
Any grade	53 (95%)	5 (100%)	27 (93%)	21 (95%)	0.800
Grade 3/4	40 (6%) (missing: 9)	4 (80%) (missing: 2)	16 (55%) (missing: 7)	20 (91%)	0.018
Number of patients requiring PLT transfusions	31 (53%) (missing: 7)	2 (33%) (missing: 1)	11 (37%) (missing: 6)	18 (82%)	0.003
Number of days with PLT transfusions, median, range	2 (1–9) (missing: 8)	1 (missing: 1)	1 (1–3) (missing: 6)	2 (1–9) (missing: 1)	0.172
The highest grade of neutropenia; number of patients, %					
Any grade	41 (76%)	3 (75%)	19 (68%)	19 (86%)	0.315
Grade 3/4	32 (59%) (missing: 11)	1 (25%) (missing: 3)	12 (43%) (missing: 8)	19 (86%)	0.003
Number of days with neutropenia, median, range					
0.5–1 × 10^9^/L	2 (0–10)	0 (0–1)	1 (0–10)	3 (0–8)	0.005
<0.5 × 10^9^/L	1 (0–7) (missing: 1)	0 (0–0)	0 (0–6) (missing: 1)	3 (0–7)	0.004
Infections:					
BSI	6 (10%)	0 (0%)	0 (0%)	6 (27%)	0.004
Pneumonia	2 (3%)	0 (0%)	1 (3%)	1 (5%)	0.863
Bronchitis	1 (2%)	0 (0%)	0 (0%)	1 (5%)	0.435
UTI	1 (2%)	0 (0%)	0 (0%)	1 (5%)	0.435
Febrile neutropenia	2 (3%)	0 (0%)	1 (3%)	1 (5%)	0.863
CVC insertion site infection	1 (2%)	0 (0%	1 (3%)	1 (9%)	0.622
URTI	1 (2%) (missing: 7)	1 (16.7%) (missing: 1)	0 (0%) (missing: 6)	0 (0%)	0.012
Number of patients with anti-infectious treatment	12 (21%) (missing: 7)	0 (0%) (missing: 1)	3 (10%) (missing: 6)	9 (41%)	0.010

**Table 4:** Factors predictive for remobilization failure in patients after previous auto-HCT. The results are presented as odds ratios (OR) along with 95% confidence intervals (95% CI) and *p*-values (CTD—cyclophosphamide, thalidomide, dexamethasone; CR—complete remission; ISS—International Staging System; VGPR—very good partial remission; PR—partial remission; SD—stable disease; PD, progressive disease).

	OR (95% CI)	*p*
Age at remobilization; as a continuous variable	0.956 (0.876; 1.044)	0.315
Age at remobilization: ≤65 vs. >65	0.316 (0.090; 1.103)	0.071
Sex: Female vs. Male	0.788 (0.227; 2.735)	0.707
Calendar year: ≤2017 vs. >2017	0.856 (0.252; 2.902)	0.802
Type of multiple myeloma: other types vs. IgG	1.489 (0.436; 5.082)	0.525
Kidney failure: ≥2 mg/dL vs. <2 mg/dL	1.714 (0.375; 7.836)	0.487
ISS at diagnosis: 1 and 2 vs. 3	3.429 (0.644; 18.259)	0.149
Number of previous lines of treatment: ≤2 vs. >2	0.912 (0.243; 3.421)	0.892
Reinduction for relapse: other vs. Bortezomib-based	1.037 (0.276; 3.898)	0.957
Multiple myeloma treatment prior to remobilization		
Bortezomib yes vs. no	1.118 (0.262; 4.775)	0.880
Alkylators yes vs. no	0.22 (0.028; 1.736)	0.151
Carfilzomib yes vs. no	2.3 (0.490; 10.787)	0.291
Thalidomide yes vs. no	0.75 (0.197; 2.849)	0.673
Lenalidomide yes vs. no	-	-
Pomalidomide yes vs. no	-	-
Bendamustine yes vs. no	-	-
Daratumumab yes vs. no	-	-
Number of mobilization attempts prior to the first auto-HCT: 1 vs. ≥2	0.769 (0.175; 3.379)	0.728
Total CD34+ × 106 cell count/kg body weight obtained prior to the first auto-HCT: ≤8 vs. >8	1.944 (0.531; 7.119)	0.315
Number of previous auto-HCTs: 1 vs. 2	0.794 (0.184; 3.428)	0.757
Total dose of melphalan before remobilization: ≤200 mg/m^2^ vs. >200 mg/m^2^	1.220 (0.230; 6.466)	0.816
Time interval between the last auto-HCT and remobilization: ≤42 months vs. >42 months	1.361 (0.402; 4.606)	0.620
Status of multiple myeloma at remobilization: ≥VGPR vs. < VGPR	1.848 (0.503; 6.785)	0.355
Status of multiple myeloma at remobilization: ≥PR vs. <PR	2.233 (0.254; 19.654)	0.469
Platelet count at the start of remobilization: ≤100 × 10^9^/L vs. >100 × 10^9^/L	6.125 (1.047; 35.824)	0.044
Myelodysplasia-related changes	No data	
Dose of cytarabine		
800 mg/m^2^ vs. 1600 mg/m^2^	4.65 (0.792; 27.301)	0.089
800 mg/m^2^ vs. 2400 mg/m^2^	2.55 (0.422; 15.406)	0.308
1600 mg/m^2^ vs. 2400 mg/m^2^	0.548 (0.139; 2.166)	0.391
800 mg/m^2^ vs. ≥1600 mg/m^2^	3.6 (0.695; 18.6460)	0.127
≤1600 mg/m^2^ vs. 2400 mg/m^2^	0.777 (0.221; 2.736)	0.695

## Data Availability

The datasets generated during and/or analyzed during the current study are available from the corresponding author upon reasonable request.

## Supplementary Materials

The following supporting information can be downloaded at: https://www.mdpi.com/article/10.3390/cancers16142588/s1, Table S1. Patients’ characteristics.
