# Supplementary material for: Stem Cell Mobilization Performed with Different Doses of Cytarabine in Plasma Cell Myeloma Patients Relapsing after Previous Autologous Hematopoietic Cell Transplantation—A Multicenter Report by the Polish Myeloma Study Group
Source: Cancers (Basel). 2024 Jul 19;16(14):2588. doi: 10.3390/cancers16142588 (PMC11274934; doi:10.3390/cancers16142588)
Supplement: Supplementary file 1 [file cancers-16-02588-s001.zip › cancers-3086744-supplementary.pdf]

cancers

MDPI

Article

Stem Cell Mobilization Performed with Different Doses of Cytarabine in Plasma Cell Myeloma Patients Relapsing after Previous Autologous Hematopoietic Cell Transplantation—a Multicenter Report by the Polish Myeloma Study Group

Joanna Drozd-Sokołowska, Anna Waszczuk-Gajda, Magdalena Topczewska, Martyna Maciejewska, Magdalena Dutka, Jan Maciej Zaucha, Anna Szmigielska-Kapłon, Mateusz Nowicki, Magdalena Olszewska-Szopa, Agnieszka Szeremet, Anna Czyż, Magdalena Kozioł, Marek Hus, Joanna Mańko, Iwona Hus, Joanna Romejko-Jarosińska, Anna Kopińska, Grzegorz Helbig, Krzysztof Mądry, Piotr Boguradzki, Małgorzata Król, Emilian Snarski, Patrick J. Hayden, Krzysztof Jamroziak, Jadwiga Dwilewicz-Trojaczek and Grzegorz Władysław Basak

Table S1. Patients' characteristics.

	Total	Cyatarabine_800	Cytarabine_1600	Cytarabine_2400	p
Number of patients	65	7	36	22	-
Type of multiple myeloma					
IgG	40 (62%)	3 (43%)	23 (64%)	14 (64%)	0.561
IgA	11 (17%)	1 (14%)	6 (17%)	4 (18%)	0.970
Non-secretory PCM	1 (2%)	0 (0%)	1 (3%)	0 (0%)	0.664
FLC κ	9 (14%)	2 (29%)	3 (8%)	4 (18%)	0.281
FLC λ	4 (6%)	1 (14%)	3 (8%)	0 (0%)	0.281
Kidney failure					
<2 mg/dL	50 (83%)	3 (43%)	28 (85%)	19 (95%)	0.006
≥2 mg/dL	10 (17%)	4 (57%)	5 (15%)	1 (5%)	0.006
	(missing: 5)		(missing: 3)	(missing: 2)	
ISS at diagnosis					
1	14 (29%)	2 (50%)	8 (27%)	4 (27%)	0.613
2	15 (31%)	0 (0%)	9 (30%)	6 (40%)	0.302
3	20 (41%)	2 (50%)	13 (43%)	5 (33%)	0.754
	(missing: 16)	(missing: 3)	(missing: 6)	(missing: 7)	
Number of lines of treatment prior to the first auto-HCT; median, range	1 (1–5)	1 (1–2)	1 (1–5)	1 (1–3)	0.815
Number of lines of treatment between the most recent auto-HCT and remobilization; median, range	1 (0–3)	1 (1–3)	1 (0–3)	1 (0–3)	0.879
Number of mobilizations attempts prior to the first auto-HCT					
1	48 (79%)	5 (71%)	29 (91%)	14 (64%)	0.052
2	10 (16%)	2 (29%)	2 (6%)	6 (27%)	0.080
3	3 (5%)	0 (0%)	1 (3%)	2 (%)	0.496
Median (range)	1 (1-3)	1 (1-2)	1 (1-3)	1 (1-3)	0.238
	(missing: 4)		(missing: 4)		
Total CD34+ x 10^6 cell count/kg body weight obtained prior to the first auto-HCT; median (range)	8.47 (1.8–46.68) (missing: 11)	13.95 (8.5–33.48) (missing: 2)	8.0 (1.82–46.68) (missing: 5)	8.9 (5.3–16.1) (missing: 4)	0.0454
Number of previous auto-HCTs					
1	52 (80%)	4 (57%)	35 (97%)	13 (59%)	0.001
2	13 (20%)	3 (43%)	1 (3%)	9 (41%)	0.001
Total dose of melphalan					
≤200 mg/m^2	51 (81%)	4 (67%)	34 (97%)	13 (59%)	0.001
>200 mg/m^2	12 (19%)	2 (33%)	1 (3%)	9 (41%)	0.001

	(missing: 2)	(missing: 1)	(missing: 1)		
Status of multiple myeloma at remobilization					
CR	11 (17%)	1 (14%)	9 (26%)	2 (9%)	0.279
VGPR	25 (39%)	3 (43%)	14 (40%)	8 (36%)	0.941
PR	18 (28%)	3 (43%)	12 (34%)	3 (14%)	0.158
SD	2 (3%)	0 (0%)	0 (0%)	2 (9%)	0.139
PD	7 (11%) (missing: 1)	0 (0%)	0 (0%) (missing: 1)	7 (32%)	0.001

auto-HCT—autologous hematopoietic cell transplantation, CR—complete remission, FLC—free light chain, IgA—immunoglobulin A, IgG—immunoglobulin G, ISS—International Staging System, PCM—plasma cell myeloma, PD—progressive disease, PR—partial remission, SD—stable disease, VGPR—very good partial remission.
